# Looking to recognise: the pre-eminence of semantic over sensorimotor processing in human tool use

**DOI:** 10.1038/s41598-020-63045-0

**Published:** 2020-04-09

**Authors:** Giovanni Federico, Maria A. Brandimonte

**Affiliations:** Suor Orsola Benincasa University, Laboratory of Experimental Psychology, Naples, Italy

**Keywords:** Psychology, Human behaviour, Cognitive neuroscience, Perception

## Abstract

Alongside language and bipedal locomotion, tool use is a characterizing activity of human beings. Current theories in the field embrace two contrasting approaches: “manipulation-based” theories, which are anchored in the embodied-cognition view, explain tool use as deriving from past sensorimotor experiences, whereas “reasoning-based” theories suggest that people reason about object properties to solve everyday-life problems. Here, we present results from two eye-tracking experiments in which we manipulated the visuo-perceptual context (thematically consistent vs. inconsistent object-tool pairs) and the goal of the task (free observation or looking to recognise). We found that participants exhibited reversed tools’ visual-exploration patterns, focusing on the tool’s manipulation area under thematically consistent conditions and on its functional area under thematically inconsistent conditions. Crucially, looking at the tools with the aim of recognising them produced longer fixations on the tools’ functional areas irrespective of thematic consistency. In addition, tools (but not objects) were recognised faster in the thematically consistent conditions. These results strongly support reasoning-based theories of tool use, as they indicate that people primarily process semantic rather than sensorimotor information to interact with the environment in an agent’s consistent-with-goal way. Such a pre-eminence of semantic processing challenges the mainstream embodied-cognition view of human tool use.

## Introduction

Tool use represents a fundamental facet of the human intrinsic ability to interact with the environment. Alongside bipedal locomotion and language, tool use is a founding characteristic of human beings. Therefore, the study of the cognitive mechanisms underlying the processing of tools is crucial in Cognitive Science. The critical relevance of the topic is well demonstrated by the large amount of research on perceptual and semantic processing of functional and motor properties of tools (also called “affordances”) that has been done in the last forty years^1^.

It should be noticed that the word “affordance” is probably one of the most ambiguous words in experimental psychology as it has acquired over time a multiplicity of meanings, hence becoming a term that generated confusion in the field of tool use, even among scholars. As an effort to reduce such an ambiguity, in this study, we endorsed the definition of the term as recently proposed by Osiurak and colleagues, i.e., as “an animal-relative, biomechanical property specifying an action possibility within a body/hand-centered frame of reference” (p. 410)^1^. Such an action possibility pertains to the physical but not to the neurocognitive domain, which, instead analyses how affordances are perceived. To this end, eye-tracking research paradigms, which investigate individuals’ gaze behaviour and their allocation of visuospatial attention, are good candidates to advance our understanding of the cognitive mechanisms associated with affordance perception^2^. Within the above framework, we used the word “affordance” or any synonym of it to refer to the action possibilities prompted by the visuo-perceptual context.

As a class of objects with intrinsic action and motor features^3–5^, tools are traditionally defined as handheld physical implementations that amplify the user’s sensorimotor capabilities. Note that, in this way, it is possible to use the word “object” in order to refer to the plausible recipient of an action^6^. However, in everyday life, tools are rarely used in isolation. They are generally part of a broader scenario that includes other objects and the set of contextual and spatial relations^7^. Thus, a large number of researchers used paradigms with paired objects (i.e., object-tool pairs) to investigate how the visuo-perceptual context modulates the functional and motor properties of tools^8,9^.

Interest in paired-object affordances has increased after the finding that visual extinction – a phenomenon in which patients with parietal damage fail to report stimuli presented on the contralesional visual field when two objects are simultaneously prompted – is reduced for objects co-located for action^10,11^. Using various paradigms, a series of studies corroborated the specificity of the paired-object affordance effect. Specifically, experiments with healthy participants investigated the cognitive and neural mechanisms underlying the facilitation that co-located-for-action and functionally linked objects provide to the perception of paired objects^8,12,13^. To date, speeded classification responses to paired objects emerge when objects are positioned in a standard co-location for right-handed actions^8^. The extraction of potential interactions between objects takes place automatically, with an affordance-related activation for objects that are “active” (for action purposes) in a visual scene and an affordance-related inhibition for “passive” objects in a visual scene^14^.

The majority of studies related to the paired-object affordance effect used compatibility paradigms to verify whether “action features” associated with an object (e.g., “graspability”) or with the visuo-perceptual context (e.g., functional or spatial relations with the objects of an object-tool pair) may have an impact on a task for which those action features are not relevant (e.g., categorization tasks). Less is known about the relative role of the visuo-perceptual context and action-related information on object recognition. Indirect suggestions come from priming studies in which a higher naming accuracy was achieved when a single object (target) was preceded by a different object (prime) with similar motor interactions. Intriguingly, the effect disappeared when the prime was a word, suggesting an action representation based on visual object information^15^. However, it has long been known that object naming and phonological retrieval rely on several distinct processes involving, among others, but not exclusively, object recognition. In turn, object recognition is based on the perception of form and colour, on visual analysis of the figure-ground relation and on the activation of stored semantic memories^16^. Therefore, object naming should not be taken as a synonym of object recognition.

From a strictly perceptual point of view, it has been suggested that an observer decodes a visual scene by incorporating functional information derived from relations between objects^17^. In particular, it appears that observers tend to perceptually group objects following familiar functional relations (e.g., a pitcher and a glass), as objects and their functional relations interact for object identification^12^. More recently, in a behavioural experiment, Borghi and colleagues^9^ used black-and-white images displaying two manipulable objects linked by either a functional (e.g., knife–butter) or a spatial (e.g., knife–coffee mug) relation. Results showed faster relatedness responses when objects were functionally rather than spatially linked.

Objects’ functional properties pertain to a kind of semantic knowledge (i.e., functional knowledge) associated with the object identity (“What is it?”) and with the goals (“What can I do with?”) attainable by using the object, whereas manipulation knowledge (i.e., sensorimotor knowledge) is related to the proper handling of an object^18^. It appears that objects’ functional knowledge can be conceived as a component of objects’ conceptual representation^19^. However, a long-established neuropsychological research tradition situates manipulation knowledge as central in tool use^20,21^. The so-called “manipulation-based” hypothesis generated strong resonance within the embodied cognition approach, which suggests that object knowledge is constituted by information inscribed within the motor and sensory systems^20–25^. In the embodied cognition perspective, it seems that the main point about tool use is to know how to manipulate it (i.e., using stored sensorimotor knowledge), rather than to reason about how the tool can be used alone or in interaction with other objects. Such a manipulation-based approach appears to be rather simplistic, especially if one considers that humans use tools to solve everyday problems, i.e., as a problem-solving situation sustained by technical reasoning skills^26^. Accordingly, recent lines of research contrasted the well-established manipulation-based approach and proposed a reasoning-based perspective whose basic assumption is that people reason about the physical object properties to solve everyday-life problems. Thus, upon seeing a tool, people do not automatically activate manipulation knowledge. Rather, they are confronted with everyday issues (e.g., hanging a picture on the wall), so that they use mechanical knowledge (i.e., technical reasoning) to reason about how to use a tool (e.g., a hammer) and solve the problem (e.g., pounding a nail in the wall). In other words, the reasoning-based theoretical framework supports the idea that people do not passively learn the relationship between objects (manipulation-based approach), but they dynamically generate it in order to “act” within a context^1,6,27–32^.

Classical neuropsychological models posit a dissociation between the visual processing streams associated with object recognition and those associated with object-directed action. It is generally accepted that the ventral stream (vision-for-perception system) is involved in object identification and recognition whereas the dorsal stream (vision-for-action system) deals with action-related and visuospatial object information mainly involved in the localization of objects in the space^33^. However, recent lines of research challenged the idea of functionally-separated processing streams for object recognition and object-directed action, assuming a joint and flexible involvement of ventral and dorsal brain areas in affordance processing^1,34–36^. In particular, as an attempt to overcome the dichotomy between manipulation-based and reasoning-based approaches, the so-called Three Action-System model (3AS) has been recently proposed^1^. On the basis of the dorsal-system partition^34^, the three neurocognitive systems of the 3AS that underlie the perception of affordances, mechanical knowledge and function knowledge are supposed to be, respectively, the dorso-dorsal system (i.e., the motor control system, in particular the bilateral superior parietal cortex and the intraparietal sulcus), the ventro-dorsal system (mainly the left inferior parietal cortex) and the ventral system (mainly the left temporal cortex). Thus, on the one hand, the reasoning-based approach to human tool use is supported by classical developmental studies that considered tool use as a problem-solving occurrence supported by technical reasoning^26^; on the other hand, the reasoning-based approach appears to be consistent with neuropsychological evidence that highlights the involvement of a wide and complex fronto-parietal and occipito-temporal brain network in tool use and affordance processing^35^.

In a most recent eye-tracking study, Federico and Brandimonte^2^, using an ecological experimental task (i.e., looking at 3D colour images depicting single tools or object-tool pairs), highlighted peculiar differences in participants’ visual exploration patterns as the degree of “action readiness” evoked by the visuo-perceptual context changed. To manipulate action readiness, the authors used thematically consistent (e.g., hammer-nail, both in the peri-personal space), thematically inconsistent (e.g., hammer-steel pot, both in the peri-personal space) and spatially inconsistent (e.g., hammer-nail, with the hammer in the peri-personal space and the nail in the extra-personal space) object-tool pairs. Results showed that single tools and tools of object-tool pairs were initially fixated longer on their functional area. However, extending the time-window of analysis, tools of thematically consistent object-tool pairs were visually encoded in a more suited-for-action way. Indeed, the fixation pattern focused on the manipulation area of the tool (e.g., the handle of a hammer) more than on its functional area (e.g., the head of a hammer). Conversely, tools of thematically and spatially inconsistent pairs obtained a reversed visuo-attentional pattern, with the functional area fixated longer than the manipulation area. It should be noticed that the experimental paradigm devised by Federico and Brandimonte^2^ involved an ecological task in which participants were asked to look at the visual scene in a natural way. Such a freely-look-at task might implicitly activate the goal of searching for potential mechanical actions between tools and objects. Hence, differences in visuo-attentional patterns might be evocative of a function-to-mechanical-to-motor “cascade” cognitive mechanism through which participants initially visually explore the scene (object-tool pairs) to gather the tool’s function knowledge (“What is it?”), then they try to solve first the mechanical knowledge issue (“How to use the tool with the object?”) and, finally, the motor control issue (“How to grasp and manipulate the tool?”). For instance, when the visuo-perceptual context is easy to decode in terms of action readiness (e.g., hammer-nail), the mechanical knowledge issue is promptly solved and the motor control instantiated, as indicated by the increase in the fixation time of the tool’s manipulation area. Conversely, when the visuo-perceptual context does not promote action readiness (e.g., bottle-cap), the mechanical knowledge issue may not be so quickly solved, so that the motor control may not be activated, as highlighted by the increment in the fixation time of the tool’s functional area. Those results were interpreted by the authors within a reasoning-based theoretical perspective, as suggesting that the flexible visuo-attentional patterns observed in the study might reflect the engagement of different tool-use neurocognitive systems (i.e., the Three Action-System)^1^. Within that theoretical frame of reference, Federico and Brandimonte^2^ introduced the concept of “action reappraisal” to refer to the cognitive processing of multiple sources of information (e.g., affordances, mechanical knowledge, functional knowledge, abstract knowledge, etc.) that can be used by an agent in order to reason about the possibility to act within and upon a context, in a proper and agent’s consistent-with-intention way^27,28^. The action reappraisal idea appears to be supported by recent neuropsychological evidence indicating the inferior parietal cortex and the middle temporal brain areas as regions where a multimodal integration of action and semantic information takes place to generate high-level cognitive representations about tools^35,37–39^.

Despite the intrinsic appeal of the action reappraisal idea, though, many questions still remain unanswered. One basic issue refers to the nature of the processing required by the task. In fact, Federico and Brandimonte^2^ used an implicit low-level task (i.e., free visual exploration of 3D images) in which the “to look at” instruction was self-sufficient for the task to be performed, with no further elaborative processing involved. However, to go a step further in the knowledge about the role of reasoning-based processing in human tool use, one should test the action reappraisal idea by introducing a kind of task that needs higher-level processing to be performed. The best candidate for such a research question is a simple short-term recognition task, which explicitly requires participants to look at the images with the higher-level purpose of recognising them as being present or not in the previously seen pair. The joint measures of visual exploration patterns and recognition performance should help disentangle the relative role of reasoning vs. manipulation-based processes. Therefore, in the present article, we investigated the effects of the action readiness prompted by the visuo-perceptual context on performance in both a lower level (free visual exploration) and a higher level (object short-term recognition) task.

We run two experiments. The first experiment was aimed at replicating the effects reported in a previous work^2^ and extending them to a new set of stimuli with a simplified paradigm. Hence, we analysed by eye-tracking the visuospatial attentional patterns of participants looking at 3D images depicting object-tool pairs that could be thematically consistent or thematically inconsistent, with the object on the left and the tool on the right, both in the person’s peri-personal space. The crucial independent variable was the thematic consistency between the stimuli composing the object-tool pairs, with the assumption that affordance perception should be facilitated in the thematically consistent condition by virtue of the higher action readiness elicited by the visuo-perceptual context^2^. In particular, we analysed the fixation patterns related to the tools of the object-tool pairs. The Areas of Interest considered in Experiment 1 were the ones related to the functional (middle-top area) and to the manipulation (middle-bottom area) parts of the tool. In accordance with previous results^2^, we expected different tool’s visual exploration patterns as the action readiness prompted by the visuo-perceptual context changed. Specifically, a longer fixation duration on the manipulation areas of the tools was expected in the thematically consistent condition.

In Experiment 2, we used eye-tracking during a yes-no short-term recognition task, in which new participants were first presented with the same object-tool pairs as in Experiment 1 (thematically consistent and thematically inconsistent pairs) and then, after each pair presentation, asked to decide whether a subsequent single object (or a single tool) was present in the original, just seen, pair. The main reason for using a short-term object recognition task was that, due to its explicit, high-level, semantic nature, such a task is instrumental in analysing differences in visual exploration patterns under object-tool thematically consistent or inconsistent conditions. In addition, a short-term recognition task should prevent participants from using recoding strategies of object-tool relations that might bias responses at test. Hence, Experiment 2 explored the novel, specific hypothesis that the activation of higher cognitive processes prompted by the goal-directed nature of the recognition task should influence the processing of the visual scene. Indeed, if the concept of action reappraisal is correct, then, contrasting a simple, spontaneous behaviour (looking at; Experiment 1) with a goal-directed behaviour (looking to recognise; Experiment 2) should make the functional-to-mechanical-to-motor cascade mechanism emerge more clearly. In particular, in Experiment 1, the activation of the implicit goal of searching for potential mechanical actions between tools and objects should produce a visuo-attentional pattern that, under higher action readiness conditions (i.e., in the thematically consistent conditions), favours the tool’s manipulation area, as a consequence of the motoric nature of the implicit task (i.e., a simpler resolution of the cascade mechanism). In Experiment 2, the higher level, goal-directed nature of the recognition task should promote functional/semantic processing over motor activations, with more fixations on the functional areas of the tools also in the thematically consistent condition. In other words, the cognitive nature of the recognition task should prevent a reasoning-based agent from proceeding toward the mechanical and then the motor processing, hence lingering in the functional/semantic processing.

Furthermore, in order to investigate the temporal dynamics of tool’s visual exploration, for both experiments we used two different time windows of eye-tracking data analysis (500 ms and 1000 ms). We expected a visual-attentional pattern initially (first 500 ms of visual exploration) focussing on the tools’ functional area in both the experiments and in all experimental conditions^2^. Finally, as regards recognition performance, in accordance with some recent literature^9,12,15,17,18,37^, faster recognition was predicted under thematically consistent conditions.

## Results

### Experiment 1

Experiment 1 was aimed to assess whether the action readiness evoked by the visual scene could modify tools’ fixation patterns. Data related to the mean fixation time spent by participants to look at the manipulation and functional AOIs of the tool, within a time window of analysis of 1000 ms, are summarized in Table 1. Data related to the first 500 ms of visual exploration are instead reported in Table 2.

**Table 1:** Experiment 1 – Mean Fixation Time of Manipulation and Functional AOIs of the Tool. Time window of analysis: 1000 ms.

		Areas of Interest (Mean Fixation Time - mean and SD)	
		*Manipulation AOI*	*Functional AOI*
Thematic Consistency	*Thematically consistent*	148.02 ms (38.87)	82.87 ms (38.18)
	*Thematically inconsistent*	68.50 ms (36.21)	139.02 ms (41.63)

**Table 2:** Experiment 1 – Mean Fixation Time of Manipulation and Functional AOIs of the Tool. Time window of analysis: 500 ms.

		Areas of Interest (Mean Fixation Time - mean and SD)	
		*Manipulation AOI*	*Functional AOI*
Thematic Consistency	*Thematically consistent*	20.36 ms (18.52)	63.8 ms (42.91)
	*Thematically inconsistent*	19.65 ms (27.97)	89.49 ms (54.57)

**Figure 1:**
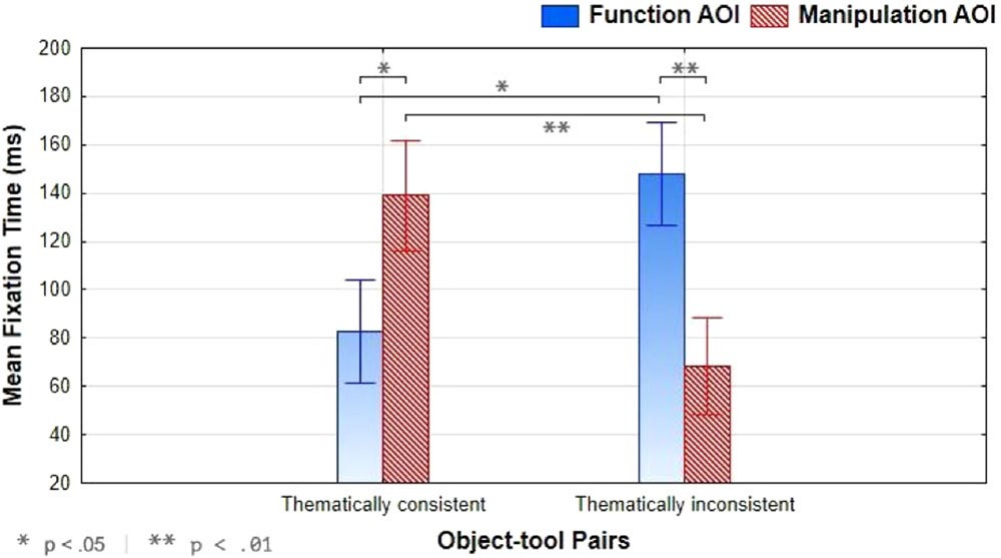
Experiment 1 – Tool’s visual exploration. In the thematically consistent condition, tools were fixated longer on their manipulation area whereas in the thematically inconsistent condition they were fixated longer on their functional area.

As regards the extended time window of analysis (1000 ms), a repeated-measure ANOVA revealed an interaction effect of Thematic Consistency and AOIs, F(1, 14) = 30.47, p < 0.001, η_p_^2^ = 0.69. Post-hoc pairwise comparisons revealed that fixation duration for the manipulation AOI was longer in the thematically consistent than thematically inconsistent condition (p < 0.01). Instead, the functional AOI was fixated longer in the thematically inconsistent than thematically consistent condition (p < 0.05). In the thematically consistent condition, the manipulation AOI of the tool was fixated longer than its functional AOI (p < 0.05), whereas in the thematically inconsistent condition the functional AOI was fixated longer than the manipulation AOI (p < 0.01). The interaction effect is shown in Fig. 1. No main effects of Thematic Consistency or AOIs were found.

As regards the initial tool’s visual exploration (i.e., with a time window of analysis of 500 ms), a second repeated-measure ANOVA revealed a main effect of the AOIs (Functional vs. Manipulation) on the tool’s fixation duration, F(1, 14) = 30.35, p < 0.001, η_p_^2^ = 0.68. The functional AOI (M = 76.65 ms, SD = 49.97) obtained longer fixations than the Manipulation AOI (M = 20 ms, SD = 23.31). No main effect of Context or interaction effects were found.

**Figure 2:**
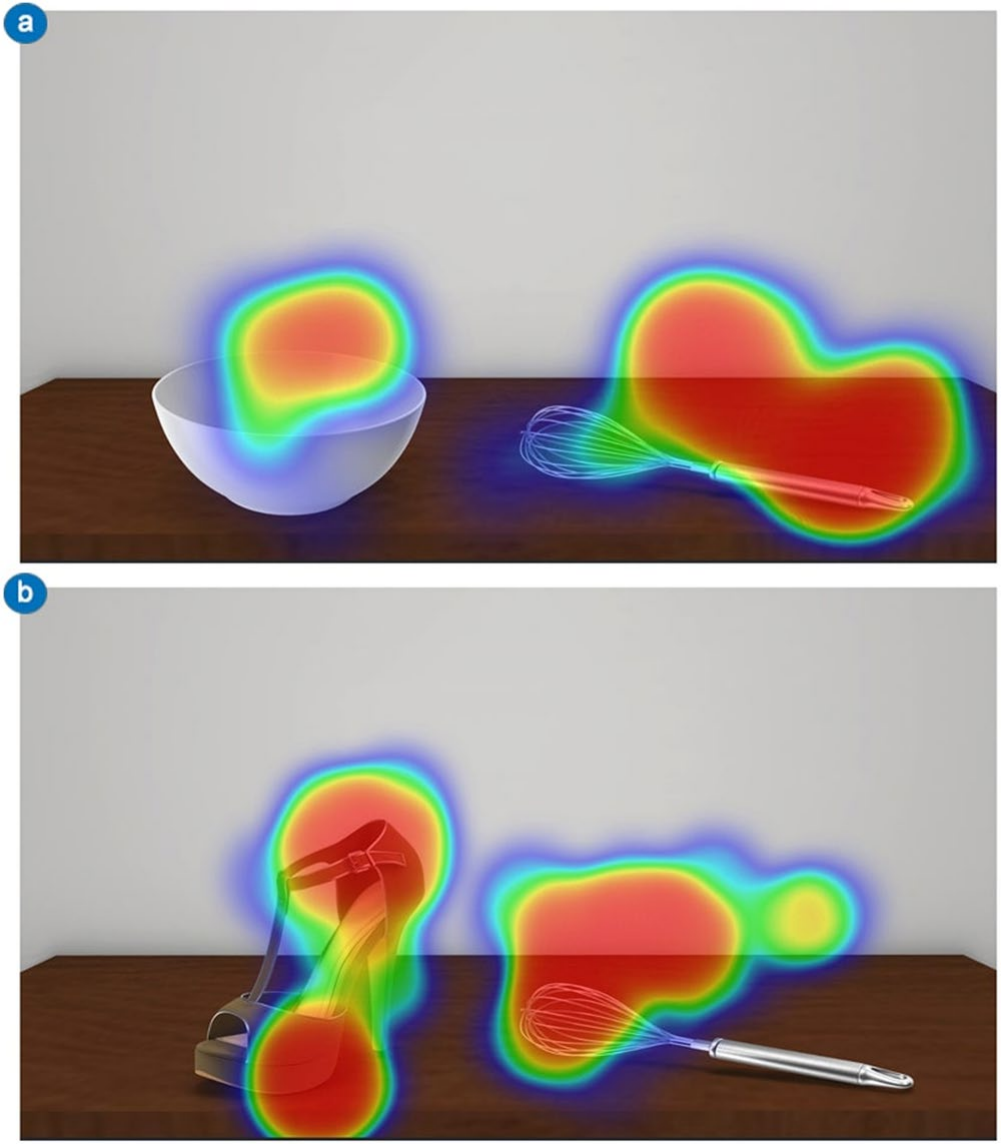
Experiment 1 – Visual exploration heatmaps. Example of heatmaps related to participants’ visual exploration of object-tool pairs used in Experiment 1. (**A**) A heatmap of a thematically consistent object-tool pair (bowl-whip) that highlights a visuo-attentional focus on the manipulable part of the tool. (**B**) A heatmap of a thematically inconsistent object-tool pair (shoe-whip) that highlights a visuo-attentional focus on the functional part of the tool. For both (**A**) and (**B**) the time window of the eye-tracking analysis was 1000 ms.

As hypothesised, participants’ visuo-attentional patterns focused on the manipulable part (e.g., the handle of a hammer) more than on the functional part (e.g., the head of a hammer) of the tools in the thematically consistent condition. Conversely, the functional area of the tools obtained more fixations in the thematically inconsistent condition (Figs. 1 and 2). Thus, tools’ specific areas obtained a reversed allocation of visuospatial attention as the action readiness evoked by the visuo-perceptual context changed. Importantly, the tool’s visual exploration started from the tools’ functional areas, as emerged from the analysis of a time window of 500 ms. These results confirm and extend previous findings regarding the emergence of peculiar tools’ fixation patterns as an effect of action reappraisal^2^.

### Experiment 2

In Experiment 2 we used a higher level, goal-directed short-term object recognition task with the aim to explore whether functional/semantic processing may take over motor activations, and how that reverberates on tool’s visual-exploration. In addition, Experiment 2 was aimed to assess differences in object recognition performance as the action readiness of the visual context changed.

#### Eye-tracking data

Data related to the mean fixation time spent by participants to look at the manipulation and functional AOIs of the tool within the extended time window of analysis (1000 ms) are summarized in Table 3, whereas data related to the restricted time window of analysis (500 ms) are reported in Table 4.

**Table 3:** Experiment 2 – Mean Fixation Time of Manipulation and Functional AOIs of the Tool. Time window of analysis: 1000 ms.

		Areas of Interest (Mean Fixation Time - mean and SD)	
		*Manipulation AOI*	*Functional AOI*
Thematic Consistency	*Thematically consistent*	72.6 ms (48.6)	217 ms (91.4)
	*Thematically inconsistent*	94.7 ms (64.5)	239 ms (86.7)

**Table 4:** Experiment 2 – Mean Fixation Time of Manipulation and Functional AOIs of the Tool. Time window of analysis: 500 ms.

		Areas of Interest (Mean Fixation Time - mean and SD)	
		*Manipulation AOI*	*Functional AOI*
Thematic Consistency	*Thematically consistent*	18.47 ms (19.69)	72.88 ms (45.5)
	*Thematically inconsistent*	20.3 ms (26.58)	91.48 ms (47.9)

**Figure 3:**
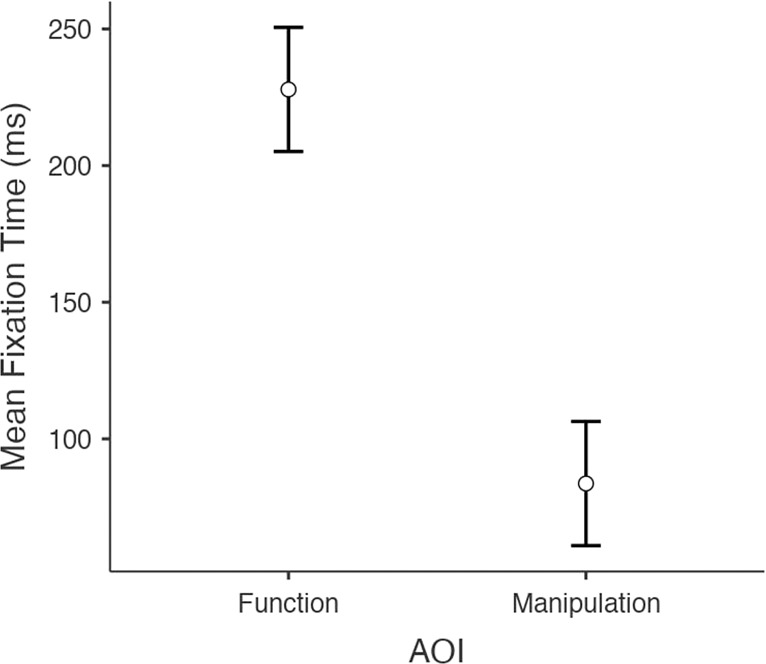
Experiment 2 – Mean Fixation Time of tool’s functional and manipulation AOIs. In Experiment 2, tools were fixated longer on their Functional AOI in all experimental condition (p < 0.001). Vertical bars denote 0.95 confidence intervals.

A repeated-measure ANOVA revealed a main effect of the AOIs on tool fixation duration within the extended time window of analysis (1000 ms), F(1, 28) = 66.62, p < 0.001, η^2^_p_ = 0.70. This main effect was due to longer tool’s fixation duration on the Functional AOI (M = 247.29 ms, SD = 107.35) than on the Manipulation AOI (M = 79.86 ms, SD = 57.37). This effect is shown in Fig. 3. A main effect of Thematic Consistency on tool’s fixation duration was also found, F(1, 28) = 5.02, p = 0.033, η^2^_p_ = 0.15. This main effect was due to longer tool’s fixation duration in the thematically inconsistent condition (M = 183 ms, SD = 117.25) than the thematically consistent condition (M = 152.78 ms, SD = 123.49). No interaction was found.

A second repeated-measure ANOVA revealed a main effect of the AOIs (Functional vs. Manipulation) on tool fixation duration within the first 500 ms of visual exploration, F(1, 28) = 82.88, p < 0.001, η_p_^2^ = 0.75. Functional AOIs (M = 82.18 ms, SD = 47.24) were fixated longer than Manipulation AOIs (M = 19.39 ms, SD = 23.2). No main effect of Context or interaction effects were found.

**Figure 4:**
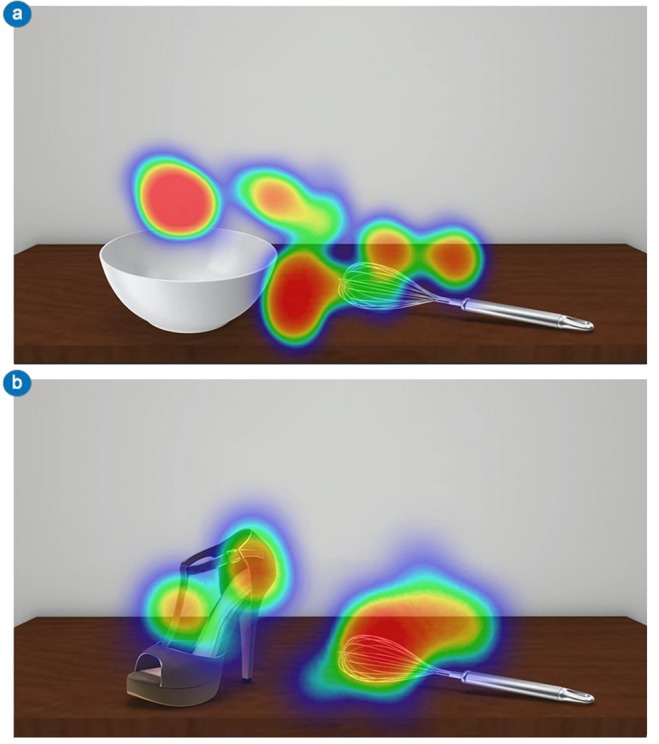
Experiment 2 – Visual exploration heatmaps. Example of heatmaps related to participants’ visual exploration of object-tool pairs used in Experiment 2. In both thematically consistent (**A**; e.g., bowl-whip) and thematically inconsistent (**B**; e.g., shoe-whip) conditions, the visuo-attentional focus was on the functional part of the tool. For both (**A**) and (**B**) the time window of the eye-tracking analysis was 1000 ms.

As predicted, these results highlight a visuo-attentional pattern that emphasises tool’s functional areas in all experimental conditions (Figs. 3 and 4). Participants’ looked at the functional part of the tools longer in both thematically consistent and inconsistent conditions. In addition, within the thematically inconsistent condition, tool’s mean fixation time significantly increased. As regards the tool’s initial visual exploration (time window of analysis of 500 ms), no differences with Experiment 1 were found, with the tools’ functional area fixated longer than the manipulation area.

#### Behavioural data

Reaction times and accuracy are summarized in Table 5.

**Table 5:** Experiment 2 – Object recognition: mean reaction times and accuracy.

Object-tool pairs	Hits		Correct Rejections	
	*Tools*	*Objects*	*Tools*	*Objects*
*Thematically consistent*	**RT (mean and SD**, **milliseconds)**			
	600 (122)	638 (107)	660 (134)	618 (115)
	**Accuracy (%)**			
	> 0.95	> 0.97	> 0.96	> 0.97
*Thematically inconsistent*	**RT (mean and SD**, **milliseconds)**			
	667 (162)	645 (138)	665 (114)	653 (132)
	**Accuracy (%)**			
	> 0.96	> 0.96	> 0.95	> 0.95

##### Hits.

**Figure 5:**
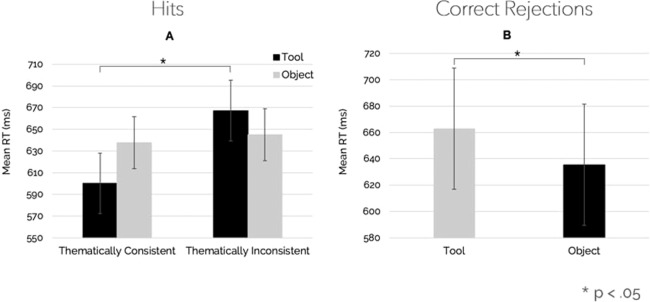
Experiment 2 – Mean RTs for Hits and Correct Rejections. (**A**) The interaction between Object Type and Thematic Consistency. Tools of object-tool pairs, but not objects, were recognised faster in the thematically consistent than in the thematically inconsistent condition. Vertical bars denote 0.95 confidence intervals. (**B**) The main effect of Object Type, with tools being rejected slower than objects. Vertical bars denote 0.95 confidence intervals.

 A repeated-measure ANOVA revealed a main effect of Thematic Consistency on RTs, F (1, 25) = 6.93, p = 0.014, η_p_^2^ = 0.22. A significant interaction between Thematic Consistency and Object Type was also found, F (1, 25) = 4.27, p = 0.049, η_p_^2^ = 0.15. Post-hoc pair-wise comparisons revealed that reaction times for tools were higher in the thematically inconsistent than the thematically consistent conditions (p < 0.05). The effect is shown in Fig. 5a. No main effect of Object Type was found.

##### Correct Rejections.

 A repeated-measure ANOVA revealed a main effect of Object Type on RTs, F (1, 25) = 5.01, p = 0.034, η_p_^2^ = 0.17. The effect is shown in Fig. 5b. No effects of Thematic Consistency or interaction were found.

The analysis of the behavioural data showed a main effect of Thematic Consistency on object recognition. When a thematically consistent pair preceded a single tool or object, participants were faster at recognising the second stimulus as part of the pair. In particular, single tools, but not objects, were recognised faster after the presentation of thematically consistent object-tool pairs (Fig. 5a). Importantly, a mirror effect of Object Type emerged for the Correct Rejections, with objects being rejected faster (Fig. 5b) than tools.

Overall, the results of Experiment 2 showed that the tool’s functional area was fixated longer irrespectively of the experimental condition. In addition, tools of thematically consistent object-tool pairs were fixated shorter and recognised faster.

## Discussion

In two experiments, we explored whether and, if so, how the visuo-perceptual context and the goal of the task may influence both the tool’s visual exploration patterns and object recognition performance. In particular, in Experiment 1, we analysed by eye-tracking the fixation patterns of tools that were part of object-tool pairs by using an implicit, low level, “looking at” visual-exploration task, while in Experiment 2 we combined eye-tracking with an explicit, higher level, “looking to recognise” short-term yes-no recognition task. By contrasting these two distinct kinds of tasks, we aimed to highlight differences in the processing of the visual scene as the action readiness elicited by the visuo-perceptual context and the goal directedness of the task changed.

The results of Experiment 1 showed that when the visuo-perceptual context prompted high action readiness (i.e., under thematically consistent conditions), a distinctive fixation pattern of the part of the tool involved in its use (i.e., manipulation area) emerged. Conversely, when the context elicited lower action readiness (i.e., in the thematically inconsistent scenes), tools were fixated longer on their functional part. The results of Experiment 1 confirm and extend previous findings^2^, by using a new set of stimuli, a simplified paradigm and a different time-window of analysis.

Recent evidence has indicated that the initial visual exploration of a tool is at least in part aimed at gaining its function, with longer fixations to the tool’s functional area (“What is it?”)^2,40^. Then, a mental simulation of the action can be produced as an effect of mechanical knowledge (“How to use it?”). Hence, visual attention shifts towards the tool’s manipulation area^1,2,27,28^. In line with these claims, when we considered a restricted time window of analysis, results highlighted a visuo-attentional pattern that focussed on the tools’ functional areas during the first 500 ms, in both experiments, regardless of the experimental conditions. This kind of function-to-mechanical-to-motor cascade mechanism is reflected in a distinctive fixation pattern reasonably generated by the interactions between functional knowledge, mechanical knowledge and motor control neurocognitive systems, as suggested by the Three Action-System model^1^. Such a flexible and dynamic mechanism pertains to a neurocognitive level of environment-information gathering that engages peculiar operational strategies, hence producing different cognitive outputs.

In Experiment 1, the looking-at task presumably activated the implicit goal of searching for potential mechanical actions between objects. Such a goal promoted a visuo-attentional pattern consistent with its implicit motoric nature. Hence, when the visuo-perceptual context suggested higher action readiness (i.e., thematically consistent condition) the simplest resolution of the cascade mechanism was reflected in a visuo-attentional pattern that emphasised the tool’s manipulation area in order to actualise the action (i.e. using the tool on the object). Conversely, the core assumption of Experiment 2 was that the higher level, goal-directed, short-term recognition task should prevent an agent from proceeding toward the mechanical and then the motor processing, hence persisting in the functional/semantic processing. Indeed, as predicted, participants exhibited a fixation pattern focused on the tool’s functional area in all experimental conditions. Additionally, in contrast with the results of Experiment 1, in Experiment 2 the tool’s mean fixation time was significantly longer under the thematically inconsistent condition. The most straightforward interpretation of this result is that thematic inconsistency made extraction of information harder^41^.

As regards object recognition performance, in Experiment 2, the analysis of the hits revealed that action-prompting tools (i.e., tools of the thematically consistent object-tool pairs) were recognised faster. Conversely, the analysis of the correct rejections showed an object-type effect, with tools being rejected slower than objects. Such an effect cannot be attributed to an alignment effect given by the spatial disposition of the objects^42^. Indeed, if that were the case, the effect should emerge also for the hits. The presence of an object-type effect only for the correct-rejection responses indicates that action and/or motoric information associated with the tools is costly in terms of rejection performance. In contrast, when the visual context stimulates the action as in the thematically consistent condition, the same tools’ action-related information improves recognition performance. Thus, a hammer is recognised faster when it is seen after a hammer-nail pair as compared to when it is seen after a hammer-scarf pair, probably because the “hammering” action possibility prompted by the visuo-perceptual context is congruent with the action-related information conveyed by the vision of the tool. In contrast, when a screwdriver is seen after the same hammer-nail pair, the “hammering” information – which conflicts with the “screwing” information prompted by the screwdriver – is reasonably no longer useful to discriminate the previous stimulus, hence producing a cost in terms of rejection performance. This process would also explain the absence of effects for the objects of the pairs.

Overall, these results suggest that action readiness and the cognitive nature of task may influence the way in which the information is collected by an observer as well as object recognition performance. These results support previous evidence in the literature that emphasised the relevance of objects’ functional relations in object recognition^9,12,15,17^, while indicating – within a cognitive theoretical framework – how an agent can utilise the set of available information in order to interact with the environment in a reasoning-based way^1,2,27,28^.

Recently, Federico and Brandimonte^2^ introduced the concept of action reappraisal to refer to a multidimensional cognitive process that utilises multiple sources of information and distinct neurocognitive systems (e.g., function knowledge, mechanical and technical knowledge, abstract knowledge, motor system, etc.) to exploit the environment in terms of action^2^. In that theoretical perspective, tools are seen as manipulable, physical implements that amplify the user’s sensorimotor capabilities^6^ in order to solve everyday problems. Tool use is thereby conceptualised as an instance of a problem-solving situation sustained by mechanical knowledge and technical reasoning^1,2,26–28,43^. Accordingly, here we endorse a theoretical approach for which individuals use tools to deal with everyday circumstances, thus reasoning about the most appropriate use of them within a context, rather than to passively learn and actualise the actions (also called “gesture engrams”) that can be performed with them (i.e., the manipulation-based approach^20–24^). This theoretical perspective is in line with the working memory hypothesis of affordances as regards the claim that the nature of the task modifies the cognitive workload and, as a consequence, affordance perception^44–46^. Furthermore, taking into account the affordance-competition hypothesis^47^ according to which various affordances are “pre-activated” before being selected, action reappraisal would be explained as a cognitive process – presumably supported by the frontal lobes – that, from the multiple environment-available affordances, selects only those that are relevant to the individual’s intentions through an inhibitory mechanism sustained by high-level executive functions^2,27^.

The assumptions underlying action reappraisal appear to be substantiated by most recent evidence revealing a multiplicity of distinct neurocognitive systems involved in tool use^1,35^. In particular, the recently proposed Three Action-System model^1^ provides a theoretical framework within which the idea of action reappraisal can be easily incorporated. For instance, the differences observed in the fixation patterns of the two experiments reported here and the facilitation effects in tool recognition found in Experiment 2 might be interpreted as reflecting the interactions between functional knowledge, mechanical knowledge and motor control neurocognitive systems. Namely, the ecological nature of the task in Experiment 1 (looking at) may have implicitly activated the cascade mechanism such that participants first solved the functional knowledge issue (“What is it?”), then the mechanical knowledge issue (“How to use it?”) and finally they activated the motor system (i.e., mental simulation of the action), hence finalizing the whole cascade process. On the other hand, in Experiment 2, the goal-oriented nature of task (looking to recognise) may have allowed participants to skip the mechanical/motor processing and to keep focused on the functional/semantic aspects of the recognition task. Coherent with this interpretation, the behavioural data revealed that the same mechanical/motor information (i.e., action readiness) evoked by the visuo-perceptual context had a facilitation effect (shorter RTs) on hits under thematically consistent conditions, but an interference (longer RTs) on correct rejections.

Mechanical knowledge (the ventro-dorsal system) might be considered as a bridge system connecting higher-level semantic information associated with object function and identity (the ventral system) and the motor-control system (the dorso-dorsal system). This bridge system would generate a simulation of an action related to tool use, hence handling the perception of the related affordances in a proper and agent’s consistent-with-intention way^2^. Such a kind of dynamic synergy among the neurocognitive systems, on the one hand, produces an effect on the way action-related information is visually encoded, coherently with the direct-visual-route-to-action theoretical view for which vision guides action;^33^ on the other hand, it substantiates how this effect might reverberate on such high-level processes as object recognition.

Recent evidence indicates that human tool use relies on a large and composite interplay of brain areas pertaining to the fronto-parietal and occipito-temporal networks^3–5,35,36,48–55^. In particular, the mechanical knowledge seems to be stored in the left inferior parietal cortex, specifically within the PF cytoarchitectonic area of the supramarginal gyrus (SMG), whereas the neurocognitive system associated to affordance perception (i.e., the motor control system) appears to be the one composed by the bilateral superior parietal cortex and the intraparietal sulcus (putative human anterior intraparietal sulcus area and the anterior dorsal IPS). The left anterior portion of SMG, extending to the PFt cytoarchitectonic area of SMG seems to be an integrative neurocognitive layer between mechanical knowledge and the motor control system^35,56,57^. Despite the neural correlates of functional knowledge are still a debated issue in the literature^49,50,58^, recent evidence indicates the left temporal cortex, the left posterior middle temporal gyrus (pMTG) and the lateral occipital complex (LOC) as plausible neural substrates of functional knowledge^35,36,52,58^.

The left inferior parietal lobule might represent a neural substrate largely implicated in forming object-related action representations. In fact, a recent fMRI study highlighted compulsory access to abstract action information in the left inferior parietal lobe for object-directed actions, irrespective of task context^53^. Coherently, in the context of tool-related action processing and in the epistemological domain of others’ action understanding^59^, a most recent meta-analysis^54^ reported the activations of both the PF cytoarchitectonic area (within the left inferior parietal lobe) and the left inferior frontal gyrus in observational tool-use contexts. As we detailed before, the PF area is involved in the storage of mechanical knowledge (i.e., the ability to reason about physical object properties). Hence, it appears that observing tools to use them and observing others’ tool-use actions share such a specific neural counterpart. These findings clearly suggest that even observing others’ tool-use actions requires supplementary cognitive skills and support the idea that tool-use action understanding might be much more “dis-embodied” than habitually supposed^54^.

In both experiments, we used object-tool pairs that were thematically consistent or inconsistent. Notably, increasing evidence from studies using thematically related vs. unrelated pairs of objects suggests an involvement of the posterior parieto-temporal cortex (specifically, the temporo-parietal junction, the inferior parietal lobe, and the middle and superior temporal gyri) in objects’ thematic relations processing^60–63^. Moreover, an increased left occipital cortex activation (ventral stream) has been reported when objects are correctly positioned for action, while the anterior regions of the dorsal stream (e.g., supplementary motor area) have been reported to be activated when the task required an action decision but objects were not in the correct position for the action^36^. Crucially, a recent TMS study has suggested that the action readiness evoked by the visuo-perceptual context facilitates the elaboration of taxonomic semantic relations among objects, indicating the inferior parietal cortex and the middle temporal areas as regions where a multimodal integration of action and semantic information takes place to generate high-level cognitive representations about tools^37^. In the same direction, a recent fMRI study used both verbal stimuli and video to assess whether specific brain areas are involved in tool-related action processing, independently of the stimulus type. By using a multi-voxel pattern analysis, that study provided compelling evidence in favour of the identification of the lateral posterior temporal cortex as a crucial brain region where a cross-modal integration of action-related information is executed. Conversely, unimodal representations produced widespread and overlapped activations in the fronto-parietal network, in the regions that were not implicated in the cross-modal integration of action-related information^39^.

The overlap of activation in the temporal and parietal brain areas reported by the above-mentioned studies appears to be particularly intriguing when analysed from the perspective of the recently proposed Hub-and-Spoke hypothesis of Semantic Memory^38,64^. As an attempt to bridge the gap between embodied and unembodied theories of conceptual knowledge (see Meteyard, Cuadrado, Bahrami and Vigliocco^65^ for a strong-to-weak embodied theories comparison), the Hub-and-Spoke hypothesis assumes that semantic memories originate from the interaction of modality-specific sources of information, called spokes (e.g., visual features, praxis or somatosensory information, sounds, etc.), with a trans-modal semantic hub that provides a further modality-invariant representational resource. Spokes rely on distributed modality-specific cortical regions (e.g., motor areas for objects’ motor properties), whereas the cortical regions within the anterior temporal region (ATL) underpin the central representational hub. The interplay between spokes and hub gives rise to consistent and generalizable concepts.

Developed in the epistemological domain of semantic memory studies, the Hub-and-Spoke hypothesis is consistent with the concept of action reappraisal as an effect of multidimensional, information-gathering processes. Following this theoretical perspective, object-related action information might be conceptualised as modality-specific motoric information that adds to the representational resources typically involved in object recognition. Importantly, such a kind of motoric information potentially emanates from some (though not all) specific spokes included in the Hub-and-Spoke hypothesis^38^.

We considered human tool use as a kind of problem-solving situation actively sustained by technical and mechanical reasoning skills^2,26–28^. In this sense, neuroimaging evidence of a left inferior prefrontal cortex involvement in action planning and execution appears to be rather promising for interpreting our results^35^. These cortical regions are extensively implicated in executive functions as well as in motor timing, sequencing and simulation^66–68^. In particular, the rostro-lateral prefrontal cortex appears to be involved in reasoning tasks such as relational integration, i.e., considering different relations simultaneously^69^. The involvement of the frontal lobes in human tool use might also signal an inhibitory mechanism that modulates the selection of the most appropriate affordance among those that are simultaneously available in the environment. Such a selection mechanism would occur in accordance with the intentions and the action possibilities of the agent^27,47^. However, given the lack of neuropsychological studies on the correlation between frontal lesions and tool-use impairments, the role of the frontal cortex in human tool use requires further investigation in future research.

To sum up, by extending previous findings^2^, the present results converge towards recent lines of research that support a reasoning-based approach to human tool use. As we detailed above, the convergence between lower-level (Experiment 1) and higher-level (Experiment 2) measures plausibly reflects the involvement of distinct and complex neurocognitive systems engaged in human tool-use processing. Although the present results do not speak directly to questions about the neural counterparts of these effects, we believe that applying the present methods to patient studies or combining them with neuroimaging procedures may help address those questions.

The concept of action reappraisal – conceived as a wide-range cognitive theoretical perspective that links together different epistemological reservoirs (e.g., psychology of perception, memory studies, neuropsychological studies, etc.) – emphasises how adopting a transversal approach might be extremely prolific to study such a human-characterizing and complex activity as tool use. It is worth noticing that our results add to the growing literature suggesting that higher-level, semantic information is activated earlier than lower-level perceptual information and can affect visual perception, although the magnitude of top down processes is modulated by the context and expectations^30–32,70,71^. To this respect, one might argue that the emphasis given in this article to semantic processing would seem in contrast with the neuropsychological literature that has shown how patients with semantic deficits can still use tools^55,72,73^. However, we examined healthy participants engaged in free-observation and object-recognition ecological tasks, rather than to evaluate tool-use tasks in clinical settings. Therefore, the results of those studies^55,72,73^ are hardly comparable to the present findings.

As we discussed, mechanical knowledge appears to be necessary to use tools (in terms of actual utilization)^1,2,6,27–29^, whereas functional/semantic knowledge might be acting as a complementary addendum in order to construct object-related action representations^12,13,15–19,30–32,39^. Thus, for patients with semantic deficits, the integrity of the neurocognitive systems associated with mechanical knowledge would guarantee for their adequate tool use abilities^55,72,73^. However, while, on the one hand, functional/semantic knowledge might be neither necessary nor sufficient in order to reason about the technical properties of a tool or to actualise the actions linked to its use, on the other hand, it might be indispensable in order to create generalizable and abstract concepts (representations) linked to tools and objects^37–39,64^. Such representations might be usable in everyday life by an agent, in the context of a cognitive-oriented functioning, as we have recently suggested. Here, we wish to emphasise the dynamicity and automaticity of higher-level cognitive processes trough which a reasoning-based agent may elaborate multiple objects-related information (i.e., action reappraisal). Hence, we investigated how the magnitude of such high-level cognitive processes may be modulated by the visuo-perceptual context and the individuals’ goals. Considering tool use as a human ability that stands at the intersection of multiple cognitive processes, the action reappraisal idea might constitute a useful starting point in order to provide broader answers regarding the mechanisms that underlie tool processing in everyday life. While recent and converging evidence seem to support the action reappraisal idea^1,2,6,27–32,35–39,44–46,53,54,59,64,71^, further studies are clearly necessary in order to explore its deepest implications.

## Methods

The experiments were conducted in the Laboratory of Experimental Psychology at Suor Orsola Benincasa University (Naples, Italy). The experiments were performed following the ethical standards laid down in the 1964 Declaration of Helsinki. The study received approval from the Ethics Committee of the Department of Educational, Psychological and Communication Sciences of Suor Orsola Benincasa University.

### Experiment 1

#### Participants

Fifteen participants (8 females; mean age = 23.07 years, S.D. = 2.19) with normal or corrected-to-normal vision took part in the experiment. All were right-handed based on the Edinburgh Handedness Inventory^74^, had no history of neurological or psychiatric disorders and gave informed consent on their participation.

#### Materials

Twenty three-dimensional (3D) computer-graphics generated stimuli were used in Experiment 1. Two different classes of stimuli were used. The first group of stimuli was composed by 3D colour images depicting pairs of objects (a tool on the right – e.g., a screwdriver – and an object on the left, e.g., a screw) that were thematically consistent, placed on the part of a table closest to the observer, in the participant’s peri-personal space. There were the following ten object-tool pairs: nail-hammer, bowl-whip, carton box-cutter, bottle-bottle opener, screw-screwdriver, salami-knife, coffee cup-teaspoon, notebook-pen, glass-bottle, padlock-key. The second group of stimuli was composed by 3D colour images depicting pairs of thematically-inconsistent objects (a tool on the right – e.g., a hammer – and an object on the left, e.g., a scarf) placed on the part of the table that was closest to the observer, in the participant’s peri-personal space. This group comprised ten object-tool pairs: scarf-hammer, women shoe-whip, alarm clock-cutter, notebook-bottle opener, nut-screwdriver, bolt-knife, Christmas ball-teaspoon, men shoe-pen, cap-bottle, baseball-key.

**Figure 6:**
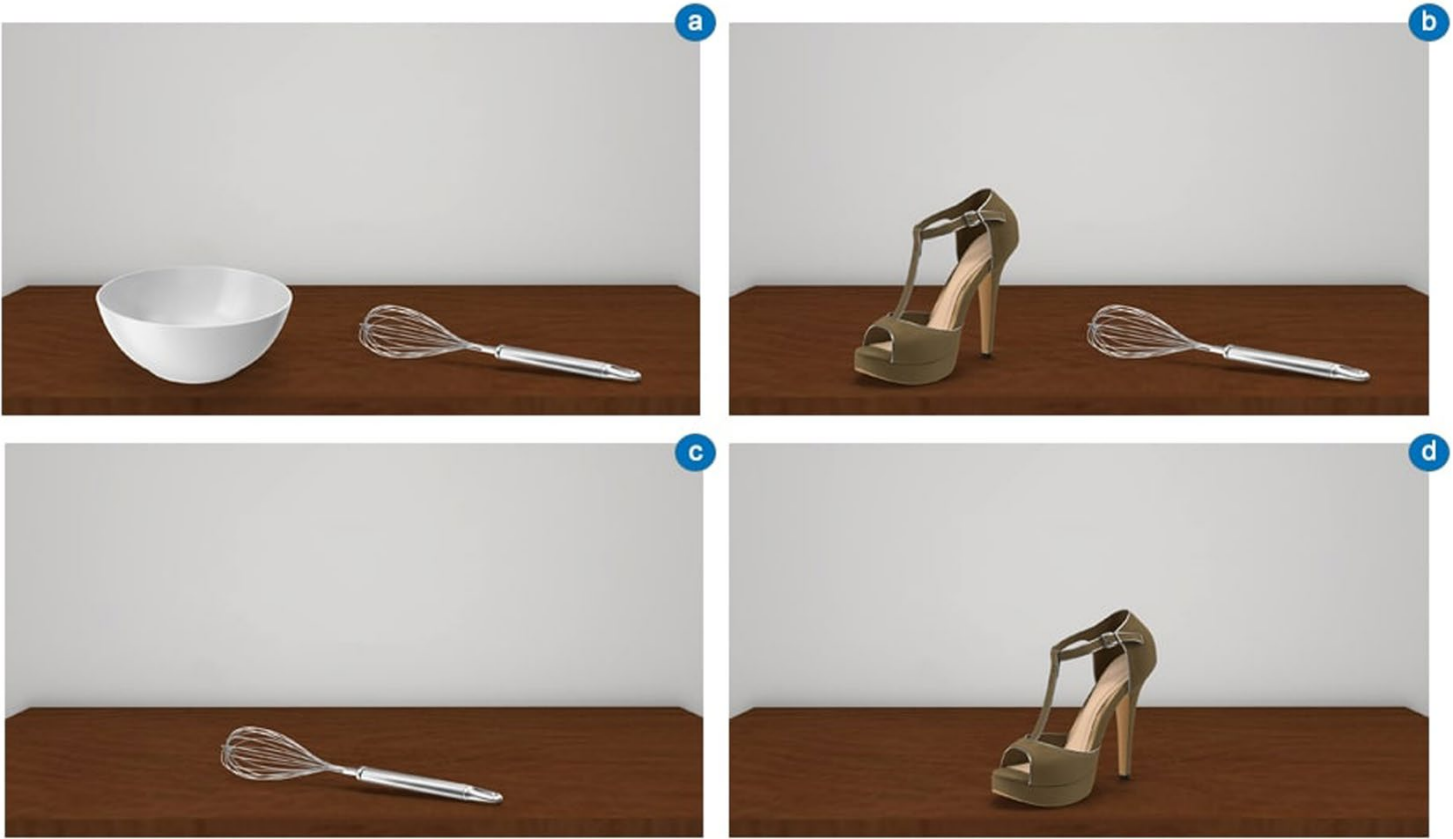
Example of stimuli used in both the experiments. (**A**) Thematically consistent object-tool pair (a bowl and a whip). (**B**) Thematically inconsistent object-tool pair (a shoe and a whip).(**C**) Single tool (a whip). (**D**) Single object (a shoe). Experiment 1 included (**A**) and (**B**). Experiment 2 included all stimuli.

Both the objects of the object-tool pairs (Fig. 6a,b) appeared placed directly on a table, with a mean perceived distance between object and tool (calculated from the centres) of approximately 20 cm and an angle of approximately 180 deg (centre-to-centre, by considering the horizontal line of the table). Some examples of the stimuli are illustrated in Fig. 6(a,b).

#### Procedure

**Figure 7:**
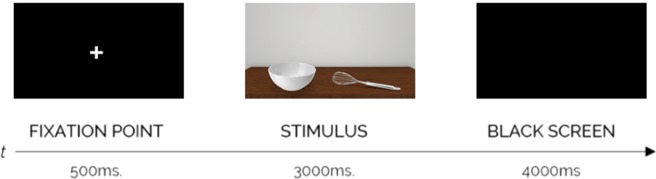
Experiment 1 - Experimental flow. For each trial, a fixation point appeared for 500 ms, then an object-tool pair appeared for 3000 ms. This pair could be thematically consistent or thematically inconsistent and it was followed by a black screen that appeared for 4000 ms to permit retina relaxation.

The air-conditioned room used in the experiment was maintained constant at a temperature of 24 °C during the entire duration of the study. Light conditions of the room were kept stable for all participants and for the entire duration of the experiment. Before starting, the participants signed informed consent. They were asked to self-report their right-handedness, their adequate visual acuity and the absence of any neurological and psychiatric diseases at the date of the experiment. The Edinburgh Handedness Inventory^74^ was administered to participants in order to verify that they were actually right-handers. Then, a classic optometric test with participants placed three meters away from the test stimuli was administered to evaluate visual acuity. The participants were seated on a chair and a headrest was used to prevent head movements in order to allow a precise eye-tracking recording. Participants seated at the distance of 54 cm from the monitor (23 inches, with a horizontal viewing angle of approximately 55 deg and a vertical viewing angle of approximately 15 deg) and were asked to keep their right hand motionless on the desk. In this way, the right hand was resting on the right side of the monitor, becoming peripherally visible to the participants in the context of the visual scene. More specifically, the right hand was located at an angle between 35 deg and 40 deg of the right visual space (mid-peripheral vision). Then, the experimental instructions were given. Participants were asked to complete an eye-tracking software calibration procedure by following with their eyes a white cross that sequentially appeared on nine parts of the screen (black background). Afterwards, participants were asked to “observe what appeared on the screen in the most natural way as possible” and the experiment started. A single trial of ten images related to each experimental condition was administered. Thus, twenty images were randomly presented according to the experimental visual flow (Fig. 7): before each stimulus, a fixation point (white cross over black background in the centre area of the screen) of 500 ms duration was shown. Then, the stimulus appeared for 3000 ms. After each stimulus, a black screen appeared for 4000 ms in order to permit retina relaxation. Each single presentation lasted 7.5 seconds (500 ms + 3000 ms + 4000 ms). Globally, the stimuli presentation lasted 150 seconds (7500 ms x 20 stimuli). At the end of the stimulation, a reachability task was administered using the same stimuli as those used during the experiment, which required participants to indicate if tools and objects in the visual scene were graspable with their right hand, according to their perspective. Participants correctly reported that both the tools and the objects were reachable with their right hand. Most relevant, in the thematically consistent condition, participants reported that the tool was potentially usable on the object (e.g., the hammer was effectively usable on the nail), whereas, in the thematically inconsistent condition, tools and objects were reported to be not immediately usable together in a proper way (e.g., the bottle opener was not considered usable on the glass despite their spatial proximity). For each participant, the overall duration of the experiment was 13 minutes. At the end of the experiment, participants were debriefed regarding the purposes of the study and the methods used. No participant was excluded from the sample.

#### Apparatus and software paradigms

We used a Full-HD Webcam (Logitech HD Pro C920, sampling rate: 30 Hz) as eye-tracking hardware and custom Python/JavaScript software to manage the experiment and to acquire gaze behaviour data. The eye-tracking technology used in the present study is based on WebGazer, an eye-tracking JavaScript library^75^. All the software paradigms were conceived and programmed using the Python programming language and the PsychoPy framework^76^ with custom code optimization. In order to extract and analyse the data collected through the software paradigms, distinct ad-hoc, custom-made scripts were engineered and developed using PHP programming language and the MySQL Database Management System. All stimuli were presented on a 23-inches monitor (Dell S2319H) at a resolution of 1920*1080px. All experimental software was executed by using an Apple MacBook Pro (13-inch, 2017) running macOS Catalina (version 10.15).

#### Gaze-behaviour data

**Figure 8:**
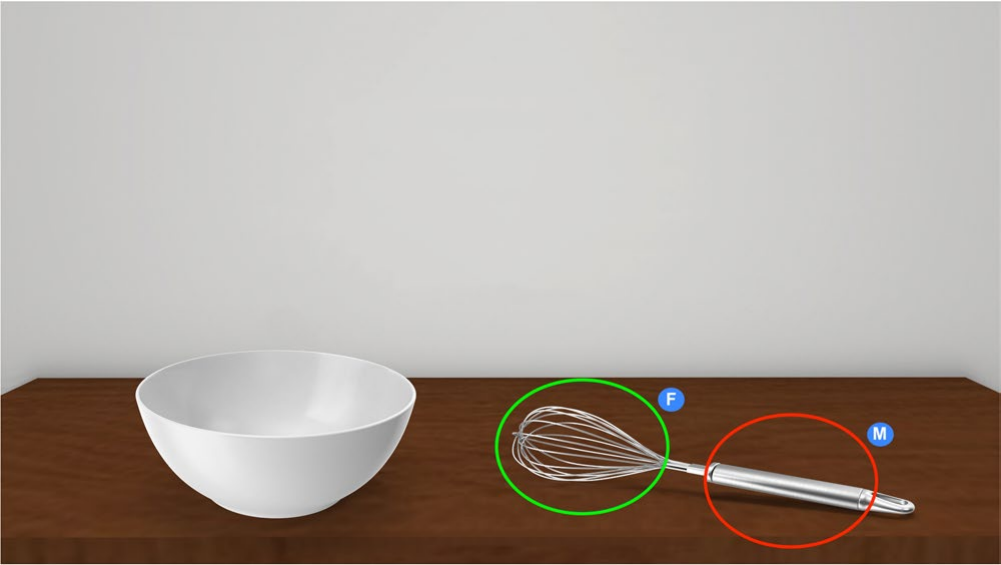
Areas of Interest considered in the experiments. The AOIs considered in the experiments were the manipulation area of the tool (circled in red, labelled as “M”) and the functional area of the tool (circled in green, labelled as “F”).

Participants’ visual-exploration patterns were analysed in terms of mean fixation duration (milliseconds) on different Areas of Interest (AOIs). We defined two AOIs: the manipulation area of the tool (i.e., the middle-bottom area where to put the hand in order to use it) and the functional area of the tool (i.e., middle-top area of the tool through which it is possible to understand its function; Fig. 8). Taking into account the technical limits of the eye-tracking technology used, both the AOIs were computed with a perimeter increased by 64 pixels in all directions. Mean fixation durations to the AOIs were averaged per condition. For each stimulus, the first 250 ms of eye-tracking data were excluded from the gaze-behaviour analysis in order to reduce the error produced by the fixation point in participants’ visual-exploration patterns. Only the first 1000 ms of visual-exploration data for each stimulus were analysed in order to reduce data dispersal effects due to participants’ visual-scene exploration. Within the first 1000 ms of visual exploration, two different time windows of analysis were considered (first 500 ms and first 1000 ms). An at-a-glance qualitative indication of differences in participants’ fixation patterns may be appreciated in the visuo-attentional heatmaps (Fig. 2).

#### Data analysis

To analyse how participants looked at the tools of the object-tool pairs as the visuo-perceptual context changed, we performed a 2 × 2 repeated measure ANOVA with AOIs (manipulation vs. functional area) as a 2-level factor and Thematic Consistency (thematically consistent vs. thematically inconsistent pairs) as a 2-level factor on tool fixation duration (milliseconds) for each time window of analysis (500 ms and 1000 ms). An alpha level of 0.05 was used for all the analyses. We used Bonferroni correction for multiple comparisons. All the analyses were conducted by using the open-source statistical software “R” (v. 3.6.1; GUI v. 1.70; build 7684) for Apple macOS operating system (Catalina, v. 10.5).

### Experiment 2

#### Participants

Twentynine participants (11 females; mean age = 21.16 years, S.D. = 3.62) with normal or corrected-to-normal vision were included in this experiment. All were right-handed based on the Edinburgh Handedness Inventory^74^ and had no history of neurological or psychiatric disorders. Participants gave their informed written consent.

#### Materials

Forty three-dimensional (3D) computer-graphics images generated stimuli were used in the experiment. Four different classes of stimuli were used. The first two groups of stimuli were the same as in Experiment 1 (10 thematically consistent and 10 thematically inconsistent object-tool pairs). The third group of stimuli was composed by 3D colour images of a single tool placed at the centre of a table in the participant’s peri-personal space. This group was composed of ten stimuli: hammer, whip, cutter, bottle opener, screwdriver, knife, teaspoon, pen, bottle, key. The fourth group of stimuli was composed by 3D colour images of a single object placed in the centre of a table in the participant’s peri-personal space. This group comprised ten objects: nail, bowl, carton box, bottle, screw, salami, coffee cup, notebook, glass, padlock. Single objects and tools (Fig. 6c,d) appeared placed on the centre of a table, with the centre of the stimulus aligned with the centre of the table. Some examples of the stimuli are illustrated in Fig. 6(a–d).

#### Procedure

**Figure 9:**
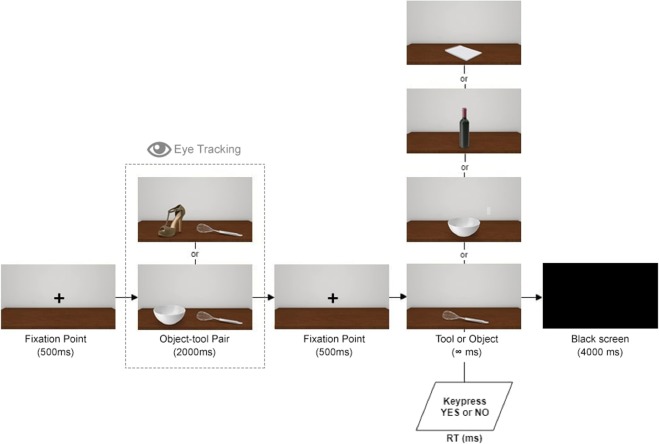
Experiment 2 – Experimental flow. A fixation point appeared for 500 ms, then an object-tool pair appeared for 2000ms. This pair could be either thematically consistent or thematically inconsistent and it was followed by a second fixation point of 500 ms. Finally, a single tool or a single object appeared. Participants pressed the “yes” key or the “no” key to indicate whether the single tool or the single object was present or not in the object-tool pair. At the end of each trial, a black screen appeared for 4000 ms to permit retina relaxation.

The experimental setting and general procedure were the same as in Experiment 1 except that participants were engaged in a short-term recognition task. Namely, participants seated at a distance of 54 cm from the monitor (23 inches, with a horizontal viewing angle of approximately 55 deg and a vertical viewing angle of approximately 15 deg) and were asked to keep their right hand motionless on the desk and the left hand on a keyboard with the index finger placed on the “E” key and the middle finger placed on the “W” key. In this way, the right hand was resting on the right side of the monitor, becoming peripherally visible to the participants in the context of the visual scene. More specifically, the right hand was located at an angle between 35 deg and 40 deg of the right visual space (mid-peripheral vision). Then, the experimental instructions were given. Participants were asked to complete an eye-tracking software calibration procedure by following with their eyes a white cross that sequentially appeared on nine parts of the screen (black background), then the experiment started. Each trial began with a fixation point (+) that remained in view for 500 ms. Then, participants had to observe a first stimulus consisting of an object-tool pair that remained in view for 2000ms. while their gaze behaviour for the stimulus was analysed through eye-tracking technology. Then, a second fixation point (+) was shown for 500 ms, followed by the target stimulus consisting of a single object or a single tool. Participants had to indicate, by pressing the appropriate key on the keyboard (“W” key for yes responses; “E” key for no responses), whether the latter stimulus (single tool or single object) was present in the previously observed object-tool pair. At the end of each trial a black screen (blank) appeared for 4000 ms in order to permit retina relaxation. Stimuli were randomly selected. The experimental paradigm was composed by a 2 (Thematic Consistency: 10 thematically consistent vs. 10 thematically inconsistent object-tool pairs) x 2 (Object Type: tools vs. objects) x 2 (Response Type: yes vs. no) within-factor design. Overall, the experiment consisted of 80 trials (10 × 2 × 2 × 2) without repetitions. The reachability task administered at the end of the experiment once again showed that participants correctly reported that both the tool and the object of the object-tool pairs were reachable using their right hand. In addition, the tool was considered usable on the object only in the thematically consistent condition. The experiment lasted approximately 30 minutes per participant. At the end of the experiment, participants were debriefed regarding the purpose of the study and the methods used as in Experiment 1. No participant was excluded from the sample. The experimental flow is summarised in Fig. 9.

#### Apparatus and software paradigms

In Experiment 2 we used the same apparatus and software paradigms as in Experiment 1.

#### Gaze-behaviour data

The same criteria as in Experiment 1 guided the analysis of data related to participants’ gaze-behaviour. Differences in participants’ visuo-attentional patterns may be qualitatively appreciated in the fixation heatmaps (Fig. 4).

#### Data analyses

First, we analysed eye-tracking data in the same way as in Experiment 1. Secondly, we analysed behavioural data related to participants’ recognition performance. Three male participants were excluded from the behavioural data analysis as their object recognition performance was above 2.5 S.D. from the mean (outliers). An alpha level of 0.05 was used for all the analyses. We used Bonferroni correction for multiple comparisons. All the analyses were performed using R (v. 3.6.1; GUI v. 1.70; build 7684) for Apple macOS operating system (Catalina, version 10.5). For all the analyses, an alpha level of 0.05 was used.

##### Eye-tracking data analysis

We performed a 2 × 2 repeated-measure ANOVA with AOIs (manipulation vs. functional area) as a 2-level factor and Thematic Consistency (thematically consistent vs. thematically inconsistent object-tool pairs) as a 2-level factor on tool fixation duration (expressed in milliseconds).

##### Behavioural data analysis

Mean reaction times and accuracy were calculated. As typically observed with this kind of recognition task, accuracy was above 0.95 in all conditions, hence we analysed only mean RTs. For the mean RTs analysis, we performed two (Hits and Correct Rejections) distinct 2 × 2 repeated-measure ANOVAs with the 2-level factor Thematic Consistency (thematically consistent vs. thematically inconsistent object-tool pairs) and the 2-level factor Object Type (tools vs. objects) on RTs.

## Data Availability

The data that support the findings of this study are available from the corresponding author upon reasonable request.

## Code availability

Software code and scripts are available on reasonable request to the corresponding author.
